# Neuroimaging and CSF Findings in Patients with Autoimmune Encephalitis: A Report of Eight Cases in a Single Academic Center

**DOI:** 10.3390/neurolint14010014

**Published:** 2022-01-28

**Authors:** Hongyan Wu, Hongxuyang Yu, Joe Joseph, Shruti Jaiswal, Shreya R. Pasham, Shitiz Sriwastava

**Affiliations:** 1 Department of Neurology, West Virginia University, Morgantown, WV 26506, USA; hongyan.wu@wvumedicine.org (H.W.); hongxuyang.yu@hsc.wvu.edu (H.Y.); 2 Department of Neuroradiology, West Virginia University, Morgantown, WV 26506, USA; jtjoseph@hsc.wvu.edu; 3 West Virginia Clinical and Translational Science Institute, Morgantown, WV 26506, USA; nickyjaiswal3@hotmail.com; 4 Malla Reddy Institute of Medical Sciences (MRIMS), Hyderabad 500055, India; p.shreyareddy25@gmail.com

**Keywords:** Autoimmune Encephalitis, cerebrospinal fluid, magnetic resonance imaging, *N*-methyl-d-aspartate encephalitis

## Abstract

Autoimmune Encephalitis (AIE) is a rare and complex group of disorders wherein the body’s immune system attacks and causes inflammatory changes in the central nervous system (CNS). It presents with altered mental status and a diverse range of typical and atypical symptoms and neuroimaging and cerebrospinal fluid (CSF) findings. The objective of this article is to highlight the importance of early identification of neurological symptoms, prompt diagnosis with neuroimaging and CSF findings, and timely management for early and complete resolution of the disease and long-term benefits. We report eight AIE cases from a single academic center confirmed by the presence of specific serum and CSF autoantibodies. The patients were mostly women, with imaging findings showing T2-weighted (T2), fluid-attenuated inversion recovery (FLAIR), hyperintensities/changes in cortical/mesio-temporal regions on a magnetic resonance imaging (MRI), and delta brush wave patterns or epileptogenic patterns on an electroencephalogram (EEG). Among the antibodies, the *N*-methyl-d-aspartate receptor (NMDA-R) antibody (AB) was most frequently identified, and CSF lymphocytosis and elevated CSF glucose were found in majority of the cases, CSF pleocytosis and elevated protein only in a minority of patients, and oligoclonal bands (OCBs) only in NMDA-R encephalitis. Early treatment with intravenous immune globulin (IVIG), steroids, plasmapheresis (PLEX), and rituximab was started in most cases, and all of them responded well and survived, but some had residual symptoms or relapses.

## 1. Introduction

Autoimmune Encephalitis (AIE) is a newly emerging category of inflammatory disorders of the brain parenchyma and surrounding structures, characterized by the presence of different antineuronal autoantibodies involving the limbic structure most commonly and the neocortex, hindbrain, striatum, spinal cord, and the peripheral nervous system [1]. AIE is a difficult clinical diagnosis due to its diverse clinical features ranging from mild or subacute deficits of memory and cognition to more complex forms of encephalopathy in the form of suppressed level of consciousness or coma with refractory seizures, along with similarities in the clinical, imaging, and laboratory findings of many forms of autoimmune, infectious, and other causes of encephalitis and, therefore, remains a diagnosis of exclusion [1,2]. Although considered to be a relatively uncommon diagnosis, Autoimmune Encephalitis is now believed to have been the differential diagnosis for a subgroup of altered mental status cases that were previously considered idiopathic [1].

In this study we reviewed laboratory findings, imaging, treatments, and outcome of eight AIE patients with confirmed serum or CSF autoimmune antibodies. We extracted and analyzed the cumulative reported frequencies and level of CSF protein, glucose, cell count, and lymphocyte percentage with respect to specific AB defined AIE subtype. The aim of this study, in the context of the overall clinical picture, is to report neuroimaging findings and CSF analysis of eight AIE patients associated with specific antibodies from a single academic center.

## 2. Case Summary

**Figure 1:**
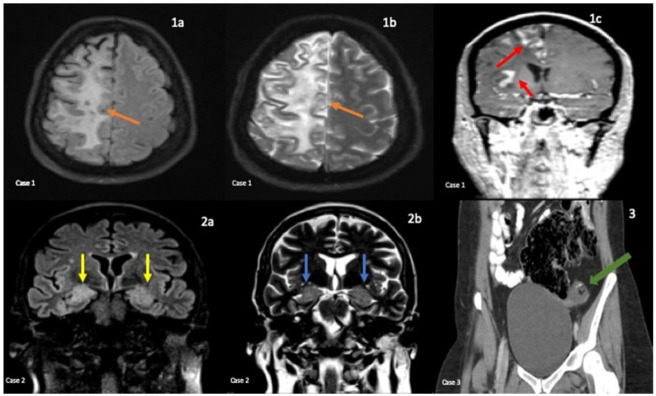
CASE 1: Axial FLAIR (**1a**), Coronal T2 (**1b**), and post contrast coronal T1 RAGE (**1c**) sequences. Multifocal areas of confluent subcortical T2/FLAIR hyperintensity (orange) with overlying gyriform cortical enhancement (red) involving the right frontal, parietal, and occipital lobes. CASE 2: Coronal FLAIR (**2a**) and T2 (**2b**) sequences. Symmetric T2 (blue) and FLAIR (yellow) hyperintensity with mild swelling in the mesial temporal lobes involving the amygdala and hippocampi. CASE 3: Coronal CT of the pelvis with IV and oral contrast (**3**). Left ovarian mass containing fat and calcification consistent with a teratoma (green).

**Case 1** was a 33-year-old woman diagnosed with Rasmussen encephalitis at the age of 27 with the initial presentation of refractory focal seizures. Her neurological exam showed mild weakness, decreased left pinprick, and right ptosis. Her MRI brain showed an extensive T2 hyperintense lesion along the right frontal cortical surface (Figure 1). The EEG showed continuous right temporal delta slowing and spike wave discharges. In addition to the positive serum N-type calcium channel AB (0.6 nmol/L), the patient was also found to have positive serum glutamic acid decarboxylase (GAD65) AB (0.07 nmol/L). The patient’s basic CSF panel showed mildly elevated nucleated cell (6/µL), glucose (54 mg/dL), and lymphocyte (82%). The CSF protein was not elevated (20 mg/dL), and Oligoclonal or monoclonal bands were not found in the CSF. The CSF viral panel was unremarkable. A positron emission tomography (PET) scan showed no malignancies. The patient was initially treated with interferon beta-1a, which made her symptoms worse. Later on, she was started on IVIG, which helped with her seizures. She continued receiving Rituximab and IVIG infusions and had good responses for a period of time; however, she continued to have seizures despite therapeutic serum anti-seizure medication levels.

**Case 2** was a 67-year-old woman diagnosed with anti-LG1 encephalitis at the age of 66, with the initial presentation of progressively worsening episodic decreased level of awareness. Her neurological exam showed no focal deficit. The MRI showed bitemporal FLAIR changes (Figure 1) and an incidental acute left basal ganglia lacunar stroke. The patient’s EEG showed right and left temporal seizures. She was found to have positive serum LGI1 antibodies, Striatal, and V-G K+ channel antibodies (6.27 nmol/L). Her basic CSF panel showed mild elevated protein (53 mg/dL) and glucose (71 mg/dL) and no cell count. The CSF viral and paraneoplastic panel were negative. Her PET scan showed no malignancies. She was treated with IVMP and oral steroids, which rapidly improved her symptoms. The initial plan was to transition to Cellcept; however, the patient continued monthly IVIG due to elevated liver enzymes. The patient has been doing fairly well.

**Case 3** was a 36-year-old woman diagnosed with anti-NMDA receptor encephalitis at age 34, with the initial presentation of acute psychosis and altered mental status (AMS). She was confused and nonverbal upon the initial examination. Her initial MRI brain and EEG were unremarkable, but a subsequent EEG two weeks later showed extreme delta brush. A left ovarian teratoma was found on the transvaginal ultrasound (Figure 1), and the patient underwent exploratory laparotomy with left oophorectomy. In addition to positive serum NMDA titers, CSF NMDA-R AB IF titer, and GFAP IFA titer (1:32 and 1:1024 respectively), the basic CSF showed elevated nucleated cell (120/µL), protein (48 mg/dL), glucose (48 mg/dL), and lymphocyte (90%). Oligoclonal bands were also found in her CSF (seven bands). She did have a positive serum Lyme disease titer, but the CSF viral panel was unremarkable. She received IVIG, intraveneous methylprednisolone (IVMP), oral steroids, and, subsequently, rituximab and showed signs of improvement after doses of rituximab. She was scheduled to receive rituximab as an outpatient but has not followed up with neurology since October 2019.

**Figure 2:**
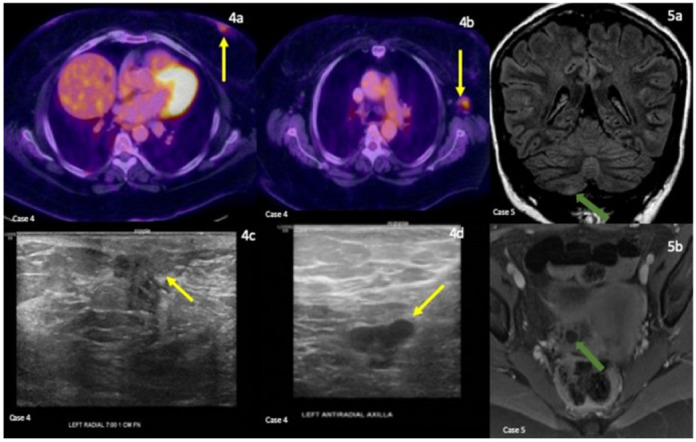
CASE 4: PET/CT of the chest (**4a**,**4b**), breast (**4c**), and axilla (**4d**) ultrasonography. Left breast subareolar hypermetabolic mass (**4a**) with hypermetabolic left axillary lymphadenopathy (**4b**) corresponding to suspicious left breast mass (**4c**) and left axillary lymph node (**4d**) on ultrasound. Coronal FLAIR (**5a**) sequence. Focal FLAIR hyperintensity in the right inferomedial cerebellum. Pelvic MRI with axial T1 fat suppressed post contrast (**5b**) sequences. Non-enhancing intrinsically T1 hyperintense right ovarian mass demonstrating fat suppression consistent with a teratoma.

**Case 4** was a 65-year-old woman diagnosed with anti-AMPA encephalitis at the age of 64, with the initial presentation of rapid progressive AMS and visual and auditory hallucination. Her neurological exam showed no focal deficit. Her MRI brain and EEG were unremarkable. In addition to the positive CSF AMPA-R AB (1:256), she was also found to have anti-thyroid peroxidase (anti-TPO) serum AB (>1000 IU/mL). The patient’s basic CSF panel showed mildly elevated protein (48 mg/dL) and glucose (52 mg/dL). There were no nucleated cells or oligoclonal bands. The patient’s mental status significantly improved after receiving IVMP and oral steroids. However, three months later, the patient was admitted again due to drastic changes in her short-term memory. She again completed IVMP, then IVIG, and rituximab. Her PET scan showed hypermetabolic activity in the thyroid gland and left breast, and the patient was later confirmed to have breast invasive ductal carcinoma with lymph node involvement (Figure 2). She underwent modified radical mastectomy and was doing well with Rituximab infusions.

**Case 5** was a 21-year-old woman diagnosed with anti-NMDA receptor encephalitis at the age of 20, with the initial presentation of headaches, seizure, and episodes of hallucinations. Subsequently, she required intubation and tracheostomy due to deteriorating mental status and respiratory failure. Her neurological exam was significant for some nystagmus and dysmetria. The initial MRI brain and EEG were unremarkable. The repeat MRI brain showed mild bilateral temporal lobe enhancement, and another followup MRI showed punctate right cerebellar lesion (Figure 2). In addition to the positive CSF NMDA-R AB IF titer, her basic CSF panel showed elevated nucleated cell (107/µL), glucose (72 mg/dL), and lymphocytes percentage (100%). OCBs were found in her CSF (four bands). The CSF protein level was not elevated (19 mg/dL) and the CSF viral panel was also unremarkable. The patient was found to have right ovarian teratoma (Figure 2) and underwent bilateral salpingo-oophorectomy. She was treated with IVMP, plasmapheresis, IVIG, and rituximab and was discharged to a rehabilitation facility. She sparsely improved and continued to have insomnia, mood changes, and short-term memory issues. Currently, she is on lacosamide for seizures and is scheduled to receive rituximab every six months.

**Figure 3:**
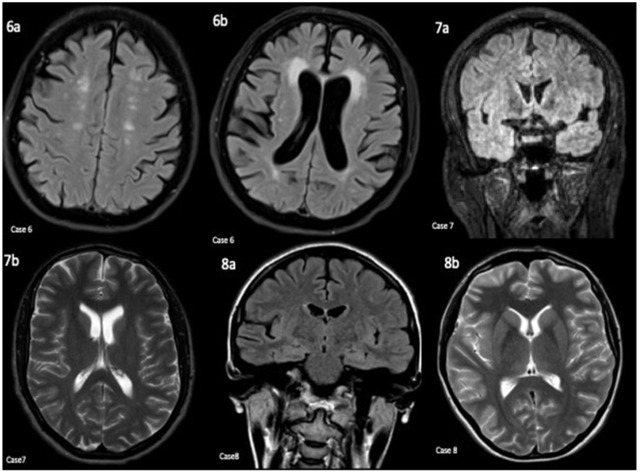
CASE 6: Axial FLAIR (**6a**,**6b**) sequences. Moderate to advanced generalized parenchymal volume loss without focal signal abnormality involving the mesial temporal lobes. There was also advanced white matter chronic microvascular ischemic changes. CASE 7: Coronal FLAIR (**7a**) and axial T2 sequence. Normal appearance of the mesial temporal lobes. The rest of the examination was also normal. CASE 8: Coronal & Axial FLAIR/T2 (**8a**,**8b**) sequence. Normal examination without any mesial temporal or other focal signal abnormalities.

**Case 6** was a 67-year-old woman diagnosed with anti-GAD receptor encephalitis at the age of 63, with the initial presentation of rapid memory decline over two months. Her initial neurological exam was only significant for bilateral distal upper and lower extremities weakness. Her MRI brain showed chronic small vessel changes (Figure 3). Her EEG showed diffused slowing without any electrographic discharges. In addition to the positive serum GAD AB titer (238 nmol/L), her basic CSF panel showed elevated CSF protein (72 mg/dL) and glucose (86 mg/dL). Her nucleated cell was 2/µL, and her CSF lymphocyte was 42%. CSF OCBs were not tested. The CSF viral panel was unremarkable. The PET scan showed no malignancies. She was treated with IVMP and, subsequently, IVIG but showed only minimal improvement. She was switched to rituximab and demonstrated some definite cognitive improvements. She was scheduled to continue receiving rituximab.

**Case 7** was a 24-year-old man diagnosed with anti-NMDA receptor encephalitis at age 23, with the initial presentation of new onset of seizures followed by personality changes. His initial neurological exam was unremarkable other than the flat affect and slowness in responding to questions. His initial MRI brain was unremarkable (Figure 3). The video EEG recorded four right/central hemispheric electrographic seizures. The patient was discharged and then readmitted two weeks later, after his NMDA came back positive. In addition to the positive CSF anti-NMDA-R AB IF titer (1:16), his serum NMDA-R AB were also positive. His basic CSF, including protein and glucose, was mostly unremarkable (22 mg/dL and 61 mg/dL, respectively). The nucleated cell was 2/µL and lymphocyte percentage was 99%. CSF OCBs were not tested. His CSF viral panel was unremarkable. The CT chest/abdomen/pelvis and scrotum ultrasound showed no evidence of malignancies. The patient was treated with IVMP and rituximab, as an inpatient, and he continued receiving rituximab after hospital discharge. The patient continued to improve, and recommendations were made for him to resume driving and to return to work in March 2021.

**Case 8** was a 24-year-old woman diagnosed with anti-NMDA receptor encephalitis at the age of 20, with the initial presentation of new onset of seizures and AMS. Her initial neurological exam and MRI brain were normal (Figure 3). Her EEG showed periodic lateralized epileptiform discharges over the right frontotemporal head regions. She was initially discharged but was re-admitted one day later due to multiple seizures and agitation. During the second admission, the patient was catatonic and did not follow commands. The patient was initially treated with IVIG, IVMP, and oral steroids with no improvement. Subsequently, the patient became febrile and showed signs of sepsis so the immune suppressant therapies were held. In addition to positive CSF NMDA-R AB IF titer (1:16), her basic CSF panel showed elevated nucleated cell (85/µL, protein (52 mg/dL), glucose (58 mg/dL), and lymphocytes (93%). OCBs were also found in her CSF (seven bands). The CSF viral panel was unremarkable. The pelvic ultrasound and pelvic MRI showed no malignancies. The patient later received IVIG, plex, IVMP, oral steroids, and rituximab and improved significantly after rituximab. This patient was noted to be “almost back to baseline” in 2019 but was lost for follow up. Chart review revealed that the patient had presented to her local emergency department multiple times for aggressive behaviors in 2019 and 2020.

## 3. Results

### Case Characteristics

A total of eight patients were included in this study. A majority of the patients were younger than 40 (5, 62.5%) and were female (7, 87.5%). Four patients (50%) had positive anti-NMDA AB, two patients (25%) were anti-GAD AB positive. One patient (12.5%) had positive anti-LG1 AB and one had positive anti-AMPA AB. Focal neurological findings (nystagmus, dysmetria, and bilateral extremity weakness) were only found in two out of eight patients (25%). Only one patient’s (case 4 with NMDA encephalitis) repeat EEG showed specific findings related to AIE (extreme delta brush). Ovarian teratomas were found in two young female patients with positive anti-NMDA AB. Invasive ductal carcinoma was found in the 64-year-old female patient with positive AMPA AB. None of the eight patients suffered fatality; however, only five patients (62.5%) were noted to be doing fairly well. The other three patients (37.5%) had some improvements but continued to have issues. Rituximab was found to be the most frequently used acute and maintenance therapy with efficacy, followed by IVIG, IVMP, oral steroids, and plasmapheresis Table 1. 

## 4. Discussion

AIE constitutes a group of noninfectious immune-mediated inflammatory disorders of the CNS, caused and diagnosed based on the presence and detection of several different neuronal autoantibodies [3]. These autoantibodies develop against synaptic proteins that are either the excitatory glutamate NMDA and AMPA receptors or the inhibitory GABA receptors, which have crucial functions in synaptic transmission and this leads to a widely variable spectrum of CNS symptoms [4]. Even though many cases go undiagnosed, AIE is still a relatively rare disease and a difficult diagnosis [1]. It affects both men and women and can occur at any age in life, but it is predominantly seen in children and young adults and in women more often than men. However, when it presents in the extremes of age, half of the patients happen to be male [2,5].

In patients with AIE the imaging and lab findings can also be quite inconsistent and recognizing characteristic findings within limbic structures suggestive of AIE is an important clue in directing its diagnosis [1]. An MRI of the brain may be normal, nonspecific, or show multifocal T2/FLAIR hyperintense signal changes [6]. The EEG has no specific pattern associations with most AIE subtypes other than extreme delta brush patterns identified in NMDA surface AB positive AIEs [7]. Such variability in findings often leads to a delay in diagnosis and treatment, which is further delayed by the prolonged time taken for AB testing [8]. This makes CSF testing crucial, as the classic CSF analysis provides more timely information indicating the inflammatory process and may support the diagnosis of AIE [9]. CSF pleocytosis has also been included as one of the clinical diagnostic criteria for AIE [10]. In addition, it helps in the classification of AIE into its various subtypes based on different characteristic CSF findings according to a systemic review study by Blinder (2019) [9].

### 4.1. Anti-Voltage Gated Calcium Channel

This subtype features extra limbic involvement with cortical enhancement and mild restricted diffusion along with subcortical T2-FLAIR hyperintensity and subsequent cortical laminar necrosis [1].

### 4.2. Anti-NMDAr

This subtype often has no imaging abnormality on initial presentation (89%) or on follow up imaging (79%) [11]. When imaging is abnormal, there can be wide variation in degree and distribution of T2-FLAIR hyperintensities, with mild cortical enhancement also possible [1]. One study demonstrated that 45% of adult women with anti-NMDAr encephalitis had an ovarian teratoma [12].

### 4.3. Anti-AMPAr

This subtype often has T2-FLAIR hyperintensities isolated to the hippocampi [13]. This subtype is most often seen in women with lung, breast, or thymic tumors [14].

### 4.4. Anti-LGI1

In this subtype, approximately 78.6% of patients demonstrate typical imaging findings of limbic encephalitis, with T2-FLAIR hyperintensity in the mesial temporal lobes. Associated restricted diffusion occurs in approximately 50% of patients, and ill-defined contrast enhancement and extrahippocampal involvement occurs in approximately 25% of patients [13].

### 4.5. Anti-GAD

This subtype is associated with classic limbic encephalitis-type imaging pattern, with swelling in the amygdala and hippocampi, which may resolve or progress to mesial temporal sclerosis [13].

### 4.6. Anti-GFAP

Perivascular radial enhancement extending from the ventricles mimicking vasculitis was seen in 53% of patients in one study [15]. Longitudinal myelitic spinal cord lesions are also a feature [15].

According to Blinder et al. (2019), the median age was 60 years or higher for patients with AIE associated with LG1 and AMPA. Patients with GAD and NMDA AB-associated AIE were considerably younger [9]. The age distributions of our AIE cases are largely consistent with the above findings. Our four patients with NMDA AB-associated AIE were diagnosed in their 20s or 30s. The patients with LG1, AMPA, and one of the two patients with GAD AB were older than 60 years of age. Past research also indicated a moderate gender dominance with certain AIE subtypes [8]; our case series did indicate a female dominance in AIE with NMDA AB (3:1 female to male ratio); however, we do not have a large enough sample to determine gender prevalence of other AIE subtypes.

CSF pleocytosis was thought to be usually present in 50% or more of the patients with NMDA and AMPA but was rare in patients with GAD and LG1 [9]. Three out of four patients with NMDA AB-associated AIE did show significant CSF pleocytosis. Pleocytosis was not present in our patients with AMPA, GAD, and LG1 AB. There was no distinct pattern with CSF protein elevations in our case series.

It has been increasingly recognized that MRI T2/FLAIR hyperintensities in the medial temporal lobes may be associated with AMPA and GAD AB AIE, and MRI imaging can return normal results for patients with NMDA AB AIE [6]. Three out of four patients with NMDA AB AIE had an unremarkable MRI brain. No patterns were discovered in other cases.

Prompt treatment and escalation of treatment in patients for whom diagnosis has been confirmed or who remain ill is associated with better outcomes [3]. Empirical treatments including IVIG, plasmapheresis, and/or steroids are often given prior to specific AB test results. Second-line treatments including rituximab or cyclophosphamide are often used in cases of first-line treatment inefficacy [5]. The treatments in our cases are consistent with the typical treatment approaches mentioned above. The majority of the patients in our case series (seven out of eight patients) were maintained on rituximab therapy after hospitalization. Three patients with carcinoma underwent surgical intervention. The majority of the patients (five out of eight patients) demonstrated definitive improvements with treatments, and there were no deaths.

## 5. Conclusions

AIE usually presents with T2-FLAIR hyperintensities or changes in cortical/mesio-temporal regions on the MRI brain. CSF changes indicating the inflammatory process are often present. Not all patients have abnormal MRI findings; early lumbar puncture with CSF analysis and AB testing is important for diagnosis in all suspected cases. CSF analysis also helps in classifying the disease etiology as well as in making therapy decisions. Early treatment yields good response and recovery in most cases.

## Figures and Tables

**Table 1:** Summary of Clinical Characteristics and Neuroimaging and CSF findings of AIE cases.

Cases	AIE Antibody	Age at Diagnosis	Sex	CSF Cell Count (/µL)	CSFPROTEIN (mg/dL)	CSF Lymph Count (%)	CSF Glucose (mg/dL)	MRI Brain	EEG Findings
1	GAD65	27	F	6	20	82	54	extensive T2 hyperintense lesion along the right frontal cortical surface	continuous right temporal delta slowing and spike and wave discharges
2	LG1	66	F	0	53	-	71	MRI brain bitemporal FLAIR changes and an incidental acute left basal ganglia lacunar stroke	bilateral temporal seizures
3	NMDA +GFAP	34	F	120	48	90	48	MRI brain unremarkable	subsequent EEG two weeks later showed extreme delta brush
4	AMPA	64	F	0	48	-	52	MRI brain unremarkable	unremarkable
5	NMDA	20	F	107	19	100	72	Intial MRI brain was unremarkable. Repeat MRI brain showed mild bilateral temporal lobe enhancement, and another followup MRI showed punctate right cerebellar lesion	unremarkable
6	GAD65	63	F	2	72	42	86	chronic small vessel disease	diffuse slowing
7	NMDA	23	M	2	22	99	61	MRI brain unremarkable	right/central seizures
8	NMDA	20	F	85	52	93	58	MRI brain unremarkable	right frontotemporal periodic lateralized epileptiform discharges

AIE, Autoimmune Encephalitis; CSF: cerebrospinal fluid; MRI, magnetic resonance imaging.

## Data Availability

This was single center case series with IRB approval Institutional Review Board at West Virginia University authorized the publication of case report, under IRB protocol number: 2006046810.

## Abbreviations

AB: antibody(ies); AIE: Autoimmune Encephalitis; CNS: central nervous system; CSF: cerebrospinal fluid; EEG: electroencephalogram; FLAIR: fluid-attenuated inversion recovery; IVIG: intravenous immunoglobulin; IVMP: intravenous methylprednisolone; MRI: magnetic resonance imaging; NMDA-R: *N*-methyl-d-aspartate receptor; OCBs: oligoclonal bands; PET: positron emission tomography; PLEX: plasmapheresis; T2: T2-weighted.
